# RisCO2: Implementation and Performance Evaluation of RISC-V Processors for Low-Power CO_2_ Concentration Sensing

**DOI:** 10.3390/mi14071371

**Published:** 2023-07-04

**Authors:** Ricardo Núñez-Prieto, David Castells-Rufas, Lluís Terés-Terés

**Affiliations:** 1 Barcelona Supercomputing Center (BSC), 08034 Barcelona, Spain; 2 Microelectronics & Electronic Systems Department, Universitat Autònoma de Barcelona (UAB), 08193 Cerdanyola del Vallès, Spain; 3 Institut de Microelectrònica de Barcelona, IMB-CNM (CSIC), 08193 Cerdanyola del Vallès, Spain

**Keywords:** RISC-V, PULPino, NDIR CO_2_ sensors, FPGA, energy efficiency, signal demodulation, power consumption

## Abstract

In the field of embedded systems, energy efficiency is a critical requirement, particularly for battery-powered devices. RISC-V processors have gained popularity due to their flexibility and open-source nature, making them an attractive choice for embedded applications. However, not all RISC-V processors are equally energy-efficient, and evaluating their performance in specific use cases is essential. This paper presents RisCO2, an RISC-V implementation optimized for energy efficiency. It evaluates its performance compared to other RISC-V processors in terms of resource utilization and energy consumption in a signal processing application for nondispersive infrared (NDIR) CO_2_ sensors.The processors were implemented in the PULPino SoC and synthesized using Vivado IDE. RisCO2 is based on the RV32E_Zfinx instruction set and was designed from scratch by the authors specifically for low-power signal demodulation in CO_2_ NDIR sensors. The other processors are Ri5cy, Micro-riscy, and Zero-riscy, developed by the PULP team, and CV32E40P (derived from Ri5cy) from the OpenHW Group, all of them widely used in the RISC-V community. Our experiments showed that RisCO2 had the lowest energy consumption among the five processors, with a 53.5% reduction in energy consumption compared to CV32E40P and a 94.8% reduction compared to Micro-riscy. Additionally, RisCO2 had the lowest FPGA resource utilization compared to the best-performing processors, CV32E40P and Ri5cy, with a 46.1% and a 59% reduction in LUTs, respectively. Our findings suggest that RisCO2 is a highly energy-efficient RISC-V processor for NDIR CO_2_ sensors that require signal demodulation to enhance the accuracy of the measurements. The results also highlight the importance of evaluating processors in specific use cases to identify the most energy-efficient option. This paper provides valuable insights for designers of energy-efficient embedded systems using RISC-V processors.

## 1. Introduction

The increasing demand for low-power and high-performance processors for embedded systems has led to the development of many architectures and implementations in recent years. In particular, the RISC-V architecture has emerged as a promising candidate for such systems due to its modular and customizable architecture. The RISC-V ISA provides an open-source and royalty-free platform that offers great flexibility in the design of embedded systems [1].

One of the most critical factors in the design of embedded systems is power consumption, which is becoming increasingly important due to the rapid growth of battery-powered electronic devices and the emergence of the Internet of Things (IoT) applications. Therefore, reducing the energy consumption of processors is a key requirement in the design of these systems [2].

This paper focuses on the design and implementation of energy-efficient RISC-V processors for use in signal processing applications, specifically in nondispersive infrared (NDIR) CO_2_ sensors [3,4]. NDIR CO_2_ sensors are widely used in various industrial and environmental monitoring applications, and their energy-efficient operation is a critical factor in their adoption.

We present a comparative study of five RISC-V processor designs in terms of energy consumption and FPGA resource utilization: RisCO2, Ri5cy, Zero-riscy, Micro-riscy, and CV32E40P. RisCO2 is an in-order, RV32E_Zfinx implementation optimized for energy consumption, designed from scratch specifically for use in NDIR CO_2_ sensors that require signal demodulation. The Ri5cy, Zero-riscy, and Micro-riscy processors are reference implementations of RISC-V processors: Ri5cy [5] is a four-stage, single-issue, in-order processor that features an RV32IMC[F] implementation that can optionally provide full support for RV32F single-precision floating-point instructions. Zero-riscy and Micro-riscy [6] are two-stage, single-issue, in-order processors with an RV32IMC and RV32EC implementation, respectively, designed to be a simplified version of Ri5cy to demonstrate how small an RISC-V CPU core could actually be. CV32E40P [7] is a four-stage, in-order, 32-bit RISC-V core derived from Ri5cy that supports the RV32IMC[F][Zfinx] instruction set.

We used the system-on-chip (SoC) platform known as PULPino [8] for implementing the five processors and simulating their energy consumption. We used Vivado 2020.2 IDE from Xilinx to synthesize, implement, and generate switching activity files to improve the accuracy of the power simulations. The results show that our design, RisCO2, is the most energy-efficient processor, consuming only 0.29 mJ of energy, which is 53.5% and 63.2% less energy than CV32E40P and Ri5cy, respectively. RisCO2 also has the lowest resource utilization compared with CV32E40P, using only 4889 LUTs, 2354 FFs, and 2 DSPs, which is 46.1%, 7.8%, and 71.4% less than CV32E40P, respectively. These findings demonstrate the effectiveness of RisCO2 in reducing energy consumption and FPGA resource utilization, making it a suitable option for battery-powered electronic devices and IoT applications.

In conclusion, the results of this comparative study demonstrate the effectiveness of custom-designed RISC-V processors in achieving energy-efficient signal processing in NDIR CO_2_ sensors. The findings show that customizing RISC-V processors can bring significant reductions in energy consumption and resource utilization compared to reference implementations, and provide insights that can guide the selection and optimization of processors for use in energy-constrained embedded systems.

## 2. Previous Work

The research on low-power RISC-V processors for embedded systems in the field of the IoT is essential because of the increasing demand for devices with extended battery life and reduced energy consumption. With the growth of IoT, there is a need for embedded processors that can perform tasks efficiently with minimal power consumption. A low-power RISC-V processor can help achieve this by reducing the device’s power consumption while still providing sufficient processing power for the intended application [9]. This can lead to longer battery life, lower costs, and more sustainable devices, which are essential in the IoT ecosystem. Additionally, the open-source nature of RISC-V makes it an attractive platform for researchers and developers who want to build low-power and energy-efficient embedded systems [10].

In that regard, there are numerous publicly available RISC-V processors with open-source RTL that cater to low-power requirements. Some of these processors are designed with a general-purpose approach, making them suitable for broad applications. Examples include VexRiscv and SweRV. On the other hand, specific applications, such as IoT, have dedicated processors such as Ri5cy, Zero-riscy, and Rocket, which are optimized for the unique demands of IoT devices.

VexRiscv [11] is a 32-bit configurable RISC-V soft processor written in SpinalHDL and developed by C. Papon in 2019. It is designed for FPGA implementation and intended for use in embedded systems and supports various configurations for custom instruction sets and peripheral interfaces. Due to its low power consumption and high performance, it is particularly well suited for FPGA-based embedded applications, such as real-time control and data processing. It has a pipeline with a configurable number of stages, from two to five stages, and provides support for the RV32I[M][F][C] instruction set.

SweRV [12] is a 32-bit, nine-stage, dual-issue, superscalar, mostly in-order pipeline with some out-of-order execution capability that supports the RV32IMC_Zicsr_Zifence ISA. The SweRV processor is intended for a wide range of applications, including storage devices, embedded systems, and data centers.

As mentioned in the introduction, the Ri5cy and Zero-riscy processors are two open-source low-power RISC-V processors designed specifically for embedded systems and IoT applications, both written in SystemVerilog by the PULP team from ETH Zurich. Ri5cy is a 32-bit, four-stage, in-order processor with a small and efficient microarchitecture. Ri5cy aims to provide a balance between performance and power efficiency, making it suitable for resource-constrained embedded systems. It supports the RV32IMC[F] instruction set, which includes the integer, multiplication, and compressed instruction set extensions.

The Zero-riscy processor is designed for ultra-low-power applications where power consumption is critical, such as IoT devices and wearable electronics. Zero-riscy features a two-stage, in-order pipeline with a small footprint and optimized power efficiency. It supports the RV32IMC instruction set and includes various power-saving techniques, such as clock gating and dynamic voltage and frequency scaling (DVFS).

Rocket [13] is an open-source RISC-V processor written in Chisel and developed at the University of California, Berkeley. A five-stage, in-order scalar core that implements the RV32G and RV64G ISA, it has a highly configurable and extensible design that serves as a platform for research and development in education and industry projects. Its open-source nature and flexible design make it a popular choice for exploring new ideas in computer architecture, prototyping novel processor features, and building custom processor designs tailored to specific applications or domains.

In line with the IoT application-specific processors mentioned above, we presented, in a previous conference paper [14], the design of a single-issue, in-order, 32-bit microprocessor utilizing the RISC-V ISA architecture intended for energy-efficient signal processing in wireless sensor nodes with a specific focus on nondispersive infrared (NDIR) CO_2_ sensors. The RISC-V microprocessor built from scratch is employed to demodulate the CO_2_ sensor signal and compute the concentration levels.

By combining various design parameters, we evaluated the performance of three processor variants to support different instruction sets, namely, RV32I, RV32IM, and RV32IMF. Our results indicate that including a floating-point unit (FPU) in the processor enhances energy efficiency in this context at the cost of an increase in hardware utilization. Specifically, adding a floating-point unit to an already optimized RV32IM design variant led to a decrease in the system’s energy consumption by more than a factor of two, although with an equivalent increase in the FPGA resource utilization of the design.

To mitigate the area increase without affecting performance, we proposed a list of improvements to be made in future work that led to the final version of our processor implementation, which we named RisCO2 to reflect its intended use in low-power embedded systems for CO_2_ concentration measurements.

In short, the improvements presented in this article encompass several aspects. Firstly, we removed the logic associated with instructions that are not utilized in the software, optimizing the design for specific application requirements. Additionally, we explored the integration of extensions to the RISC-V ISA that are better suited for embedded applications, further enhancing the processor’s capabilities. By integrating the processor into the PULPino platform, we were able to conduct comprehensive evaluations and comparisons with other reference RISC-V processors, ensuring a thorough analysis of its performance. Moreover, we conducted an extensive power breakdown analysis to precisely assess energy consumption.

For a more comprehensive understanding of the implemented improvements and their impact on processor performance, please refer to the sections below.

## 3. Proposed Architecture

We made several improvements to our previous RISC-V RV32IMF processor, resulting in the development of RisCO2. The following is a list of the actions that were implemented in successive design steps:

1. Support for the “E” extension, which halves the number of integer registers from 32 to 16 registers.
2. Support for the “Zfinx” extension, which eliminates the need for a separate floating-point register file and enables sharing of the integer register file for both floating-point and integer data. Overall, as a result of these optimizations, the register file is reduced by a factor of 1/4 compared to an RV32IMF implementation.

Once the E_Zfinx ISA was implemented, the application was compiled, and the executable .elf file was analyzed using a Python script [15]. This script searched for the number of occurrences of each ISA opcode within the compiled program. The analysis revealed that several instructions were unused, including multiplication, division, shifts, comparisons, and instructions for reading and writing in the control and status registers. Consequently, the decoder was modified in several design steps to eliminate the logic associated with those unused instructions.

3. Remove the logic associated with integer multiplication and division mul, mulh[u|su], div[u], and rem[u]. This implies that we have effectively removed the “M” extension from the implementation. This decision aligns with the application-specific nature of our design.
4. Remove the logic associated with the management of control and status registers csrrw[i], csrrs[i], and csrrc[i], as well as the unused instructions related to shifts and comparisons sra, slti, and slt.
5. Remove the logic that supports misaligned memory accesses, resulting in a simplified load-store unit that assumes all memory addresses generated by the compiler are aligned to a 4-byte boundary.

**Figure 1:**
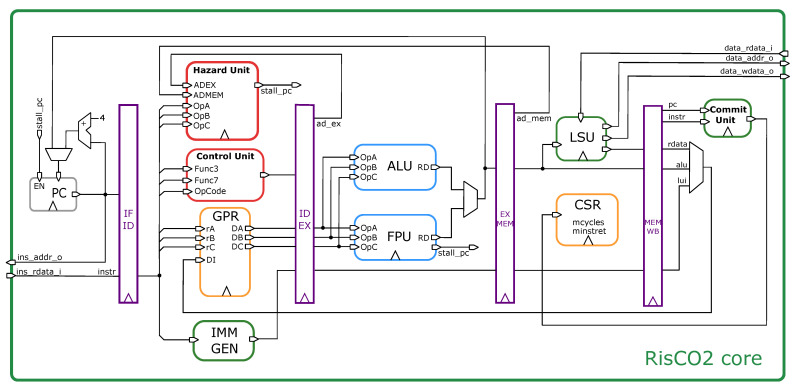
Simplified block diagram of the RisCO2 core architecture showing its five pipeline stages and all functional blocks.

The ultimate version of RisCO2 resulted in a five-stage, single-issue, in-order processor based on the RV32E_Zfinx instruction set, with a specific focus on energy efficiency. This processor is intended for use in NDIR CO_2_ sensors that necessitate signal demodulation to infer the gas concentration. The improvements made in the design of RisCO2 resulted in a reduction in energy consumption when compared to the original design, and the results are presented in Section 4. The simplified block diagram of the core is shown in Figure 1.

The RisCO2 pipeline consists of five stages through which instructions pass during execution. The stages are Fetch, Decode, Execute, Memory, and Writeback. In the Fetch stage (IF), the instruction is fetched from memory and placed in an instruction register.

In the Decode stage (ID), the instruction is decoded in the control unit, and the operands are identified and read from the general purpose register file (GPR). A hazard unit detects and resolves hazards that can occur due to dependencies between instructions. It inserts pipeline bubbles or forwards data from one stage to another to ensure that instructions are executed in the correct order and without errors.

In the Execute stage (EX), the ALU performs basic integer addition/subtraction arithmetic and logic operations, and the floating-point unit (FPU) performs addition, subtraction, multiplication, division, square-root, and fused multiply-add operations on floating-point numbers. The FPU is an open-source parameterized IP named FPnew [16], developed by the Digital Circuits and Systems Group at the ETHZ (PULP Platform). The FPU handles single-precision (32-bit) and adheres to the IEEE 754 standard for floating-point arithmetic. The unit exhibits varying latency based on the type of operation, often spanning multiple cycles. To ensure correct program execution, the unit incorporates an output to stall both the program counter (PC) and the pipeline.

In the Memory stage (MEM), data are read from or written to memory through the load store unit (LSU). The control and status register unit (CSR) contains only two registers, namely, mcycle and minstret, which are utilized for performance measurements.

In the Writeback stage (WB), the operation results are written back to the registers. The commit unit verifies when an instruction has reached the last stage of the pipeline, and its output is used to increment the minstret counter in the CSR.

### Test Methodology

The processor was implemented on a Nexys-4 Xilinx development board, featuring the XC7A100T-1CSG324C FPGA device (28 nm node), with 4860 Kib block RAM, 63,400 LUTs, 126,800 FFs, and 240 DSPs. We used Xilinx Vivado 2020.2 IDE (HLx edition) to synthesize, simulate, and estimate the power consumption of the design.

The processor was initially implemented as a standalone device in the FPGA, with direct connection to a scratchpad memory built from BRAM blocks configured as true-dual port memory. This scratchpad memory stores both the program and data.

Later, we integrated the processor into PULPino, a reference platform developed by the PULP team, which is an open-source single-core RISC-V SoC built for the Ri5cy and Zero-riscy cores. The PULPino SoC has separate single-port data and instruction RAMs and includes a boot ROM with a boot loader capable of loading a program via SPI from an external flash device. The primary objective of integrating RisCO2 into the PULPino SoC was to compare its performance in terms of resource utilization and power consumption with other RISC-V processors.

In order to accomplish this objective, we developed individual projects for each of the reference processors, namely, Zero-riscy, Micro-riscy, Ri5cy, and CV32E40P. This approach allowed us to test and evaluate the distinct integrations independently, facilitating a comprehensive comparison of the resulting outcomes.

**Figure 2:**
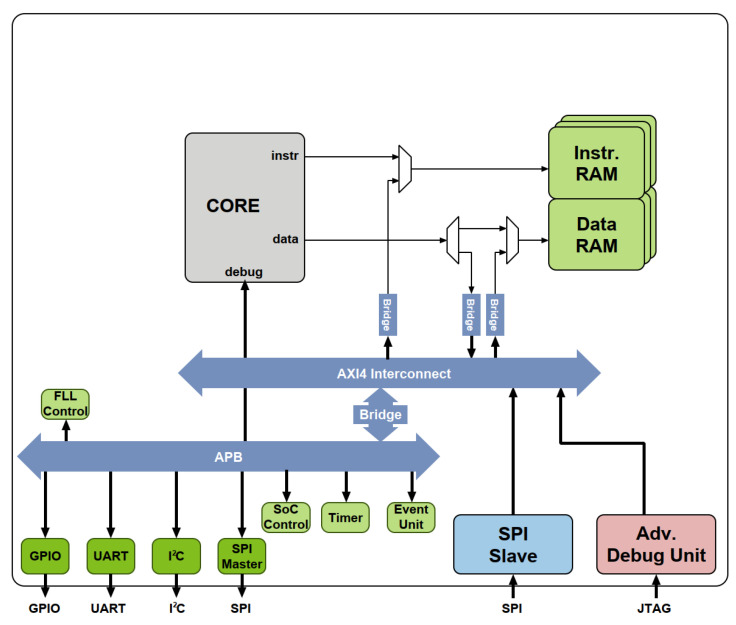
Block diagram of the modified PULPino SoC used to test the different cores.

There are several important considerations concerning the implementation of the PULPino SoC platform. Firstly, the two 32 kB single-port data and instruction RAMs were merged into a single 64 kB BRAM true dual-port memory utilizing a unified address space for both data and instruction program. Secondly, the boot ROM was eliminated from the design. Figure 2 depicts a block diagram of the PULPino platform that was customized for our testing purposes.

In the test program for our performance comparison, we utilized a for-loop-based algorithm that demodulates the digital data from the CO_2_ sensor and computes its concentration in ppm.

Notably, we did not use a physical gas sensor; instead, we opted to generate the data using a Python script. The script synthetically modulates the signal from the sensor and samples it at a rate of 16.38 kHz. Once the data were generated, we preloaded the samples in the data memory to be used in our study. Additionally, to enhance the compiled program’s performance in Zero-riscy and Micro-riscy, we utilized a C++ template class written by Schregle [17] to emulate fixed-point support since these processors lack a hardware floating-point unit. As demonstrated in [14], this approach improved the program’s execution performance.

Before synthesizing the design, we conducted RTL simulations and compared the results with data obtained from Segger Embedded Studio (SES), a commercial RISC-V ISA simulator [18]. SES serves as a development environment for open RISC-V architecture-based devices, offering a comprehensive solution for custom application development and debugging.

**Figure 3:**
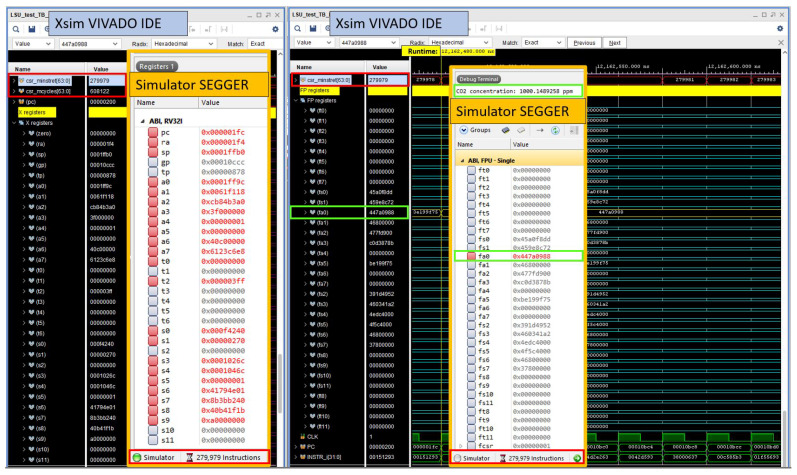
Image composite made with screenshots of the simulations performed with Vivado to test the RTL logic and Segger Embedded Studio to simulate the compiled RISC-V application.

Figure 3 shows a composite image created by overlaying screenshots from the Vivado simulator (Xsim) and the SES simulator. This simulation corresponds to the RV32IMF implementation listed in the first row of Table 1, and the figure enables thorough comparison and validation of the RTL operation. In the Segger simulator, a total of 279,979 instructions were executed, perfectly aligning with the core’s CSR counter of retired instructions csr_minstret. Furthermore, the values stored in the integer registers (depicted on the left side of the picture) and floating-point registers (on the picture’s right side) exhibit precise correspondence between both simulations.

The debug terminal in SES provides the expected output, including the calculated CO_2_ value of 1000.16 ppm, which is loaded in the fa0 register in IEEE754 format (value 0x447a0988). The simulation runtime in Vivado was 12.16 ms, utilizing a frequency of 50 MHz, as specified in Table 1. To obtain the instructions per cycle (IPC), the csr_minstret value is divided by csr_mcycles. Comparing the data obtained from both simulations validates the precise functioning of the RTL design.

Once the RTL simulation matches the RISC-V application simulation, we can proceed to synthesis and implementation and annotate FPGA resource utilization for each design version.

For synthesis, we utilized the default strategy provided by Vivado, and for implementation, we opted for the Performance_ExplorePostRoutePhysOpt strategy, which enables the physical optimization step and incorporates various algorithms for optimization, placement, and routing to potentially enhance the outcomes. With a clock frequency setting of 25 MHz, the different designs always met the timing constraints.

The dynamic power consumption of complementary metal–oxide–semiconductor (CMOS) circuits is usually expressed by Equation (1), where $C_{i}$ is the effective capacitance of the transistor *i*, *f* is the switching frequency, *V* is the supply voltage, and $S_{i,t}$ is the number of transistor swings from voltage levels of transistor *i* in time *t*. On many occasions, a simplified equation is used by collecting the total effective capacitance of the circuit *C* and a probabilistic switching activity factor α.

(1) $$P_{dyn}=\sum_{i,t}S_{i,t}C_{i}fV^{2}\approx \alpha CfV^{2}$$

In our case, we are interested in measuring total energy consumption, given by Equation (2), where $P_{sta}$ is the static power consumption.

(2) $$E=\int_{t}P_{dyn}+P_{sta}$$

To obtain a detailed estimation of $P_{dyn}$ and *E* we need to obtain the switching activity of all the gates of our circuit when executing our application. Xilinx Vivado allows capturing this activity in post-implementation timing simulation, generating switching activity files (SAIF) that are used to provide detailed power estimates for different regions of the FPGA fabric. For certain parts of the design, Vivado can obtain an accurate switching activity, but for others (such as memories), it still must use a probabilistic approach to estimate power consumption.

Capturing the switching activity for the entire demodulation algorithm runtime is unnecessary. Instead, we set the simulation interval to 1 ms, which allows us to capture multiple iterations of the demodulation algorithm’s main loop. This time setting provides a fair power average value that can be extrapolated to the entire program execution, as over 95% of the program runtime occurs within the demodulation loop.

Moreover, the power simulation tool could annotate more than 92% of the nets in all the different implementations of the SoC, performing probabilistic computations for the remaining nets. Using this methodology, we obtained accurate power estimates for each of the five RISC-V processors implemented in the PULPino SoC, providing insight into their respective energy efficiency and resource utilization.

## 4. Results

As mentioned in Section 2, RisCO2 is the result of a list of improvements applied to an RISC-V processor previously developed by the authors and presented in [14]. The list of such modifications is detailed in Section 3, and they led to a further reduction in resource utilization and consumed energy compared to the initially proposed processor.

The result of applying those actions is summarized in Table 1 step by step, together with the overall reduction in resources and consumed energy. This table shows the result of the improvements made in RisCO2 and enumerated in Section 3.

For each applied action, the table shows the FPGA resource utilization, the time it takes for the demodulation algorithm to complete, and the total energy consumed by the processor.

**Figure 4:**
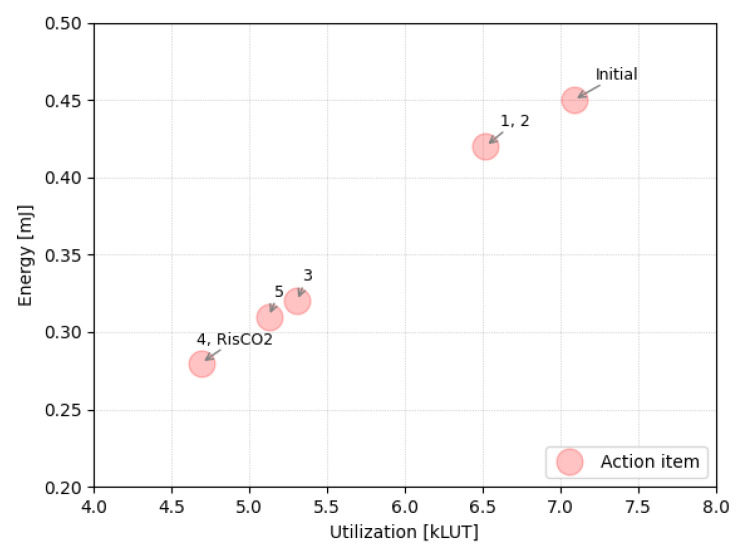
The outcome of implementing the action items enumerated in Section 3 can be observed in the correlation between the FPGA’s LUT utilization and the energy consumption of the core.

The graphical representation in Figure 4 depicts the incremental performance improvements achieved through the individual actions outlined in Section 3. The plot demonstrates a nearly proportional relationship between energy consumption and the utilization of LUTs in the design.

Table 2 compares the performance of five different RISC-V processors, including RisCO2, Zero-riscy, Micro-Riscy, Ri5cy, and CV32E40P. The comparison is based on FPGA resource utilization (LUT, FF, DSP), the number representation used by the application algorithm (fixed-point or single-precision floating-point), the number of instructions (#instr. ×10^6^) that it takes for the program to demodulate the signal and calculate the CO_2_ concentration, instructions per clock cycle (IPC), the time it takes for the demodulation algorithm to complete, and the total power and energy consumed by the processor. The clock frequency used for the comparison is 25 MHz.

RisCO2 outperforms the other processors in terms of energy consumption, with a 53.5% and 63.2% reduction in energy consumption compared to the best-performing ones, CV32E40P and Ri5cy, respectively. Additionally, RisCO2 has a lower FPGA resource utilization compared to these two. In contrast, Micro-riscy has the lowest resource utilization among the five processors, and power consumption as low as RisCO2. Still, because of the much longer execution time for the same algorithm, it has a significantly higher energy consumption than RisCO2, more than 19 times higher. The reason for the increased execution time in Micro-riscy is clearly the lack of functional units that perform more complex arithmetic operations beyond addition and subtraction. On the other hand, Ri5cy has the most increased resource utilization and consumes 2.7 times more energy than RisCO2, although it has a 50% better performance in terms of instruction throughput (IPC).

**Figure 5:**
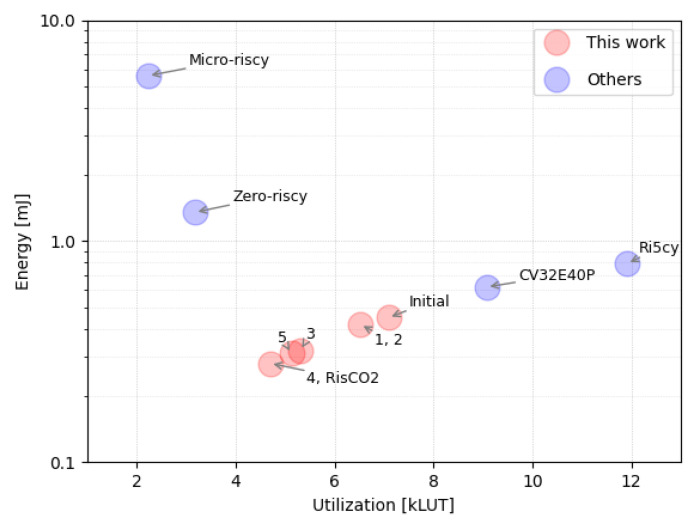
LUT utilization and energy consumption of the different cores tested in this study.

Figure 5 presents a plot similar to Figure 4 but includes the reference processors examined in our study. Notably, RisCO2 occupies a highly advantageous position within the design space, combining the strengths of both worlds. On the one hand, it shares similarities with Micro-riscy and Zero-riscy processors, characterized by minimal resource utilization and a focus on low-power consumption. On the other hand, RisCO2 draws from the strengths of Ri5cy and CV32E40P, which prioritize achieving maximum performance within a limited power budget.

A clarification is in order regarding the graph, as it shows that the initial design version of RisCO2 is already more efficient than Ri5cy or CV32E40P. This is because that version uses the most DSP blocks (12) compared to the other reference processors, which reduces the use of LUT and FF resources and leverages the specialized DSP48 blocks provided by the Xilinx FPGA, which are also more power-efficient.

**Figure 6:**
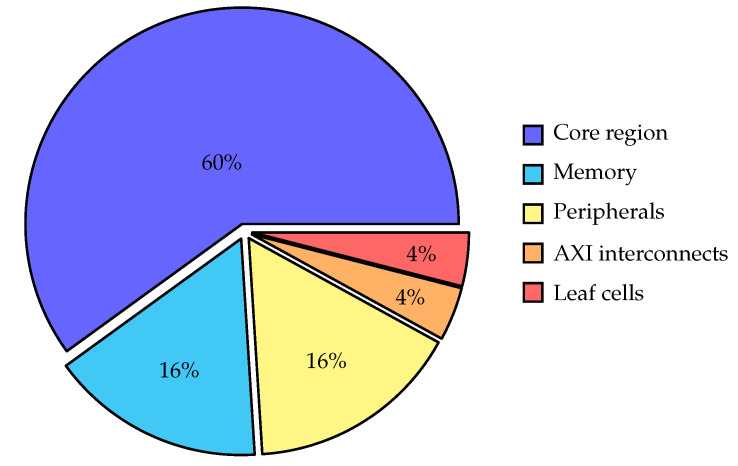
Power distribution among various components of the PULPino platform when integrating an RisCO2 processor.

The power report provided by Vivado, which is also used in Table 2, offers an estimation of the overall power consumption for the entire system. Additionally, it provides a detailed power breakdown for each of the different RTL modules in our design. Figure 6 presents a segmented pie chart that illustrates the power distribution among the various components of the PULPino platform when integrating the RisCO2 processor and executing the demodulation test program. The overall power consumption of the PULPino SoC, as reported by Vivado, is 31 mW. The breakdown of power consumption, grouped by component, is presented in the chart.

The core region consumes 60% of the total power and corresponds to the system components depicted above the AXI interconnect in Figure 2, where the processor occupies the prominent role, but excludes the instruction and data memory, which are represented separately. The processor and the memory alone account for 76% of the total power consumption.

The peripherals in the PULPino platform consume 16% of the power, although the test program does not intensively use them. They serve the purpose of extending the system’s capabilities and facilitating connectivity. They are designed to interface with the processor core, enabling seamless communication with external devices such as sensors, actuators, memory, and communication interfaces. Meanwhile, the AXI interconnects, which facilitate efficient communication and data transfer between the processor, peripheral modules, and other system components, consume as little as 4%. Additionally, another small 4% of power consumption corresponds to the leaf cells responsible for crucial functionalities related to interfacing the FPGA with external devices and ensuring optimal signal integrity.

**Figure 7:**
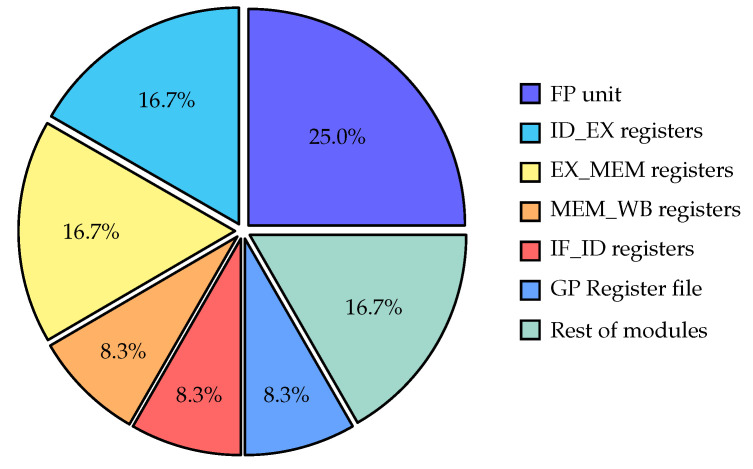
Power distribution among various modules of the RisCO2 processor.

The pie chart depicted in Figure 7 presents the distribution of power consumption among different modules of the RisCO2 processor during the execution of the demodulation test program, and the power breakdown analysis reveals interesting insights.

The floating-point unit (FPU) stands out as the most power-hungry component, accounting for 25% of the total power consumption. That is expected since the FPU performs complex floating-point operations that typically require more computational resources and power. The pipeline stage registers also contribute significantly to power consumption. The aggregated power consumption of all the pipeline stage registers in RisCO2 is 50% of the total. These registers play a crucial role in the processor’s instruction execution pipeline, facilitating the flow of data and control signals between different stages. Their relatively high power consumption can be attributed to the need for fast and efficient data transfer within the pipeline. The GP register file, which contains the processor’s general-purpose registers, accounts for 8.3% of the power consumption. That indicates that the register file, although essential for storing data during program execution, consumes less power than other critical components.

Lastly, the remaining modules collectively consume 16.7% of the power. This category includes various auxiliary circuits, control logic, and other supporting components necessary for the overall functionality of the processor.

Understanding the power breakdown helps identify the power-intensive areas of the processor design. It provides valuable insights for optimizing power consumption, such as implementing power-saving techniques in the FPU, optimizing data flow in the pipeline registers, or exploring alternative register file designs to reduce power consumption further.

## 5. Discussion

The study presented a comparative analysis of five RISC-V processor designs in terms of energy consumption and FPGA resource utilization. The processors compared were RisCO2, Ri5cy, Zero-riscy, Micro-riscy, and CV32E40P. RisCO2 is a 32-bit, in-order processor that supports the RV32E_Zfinx instruction set. It is optimized for energy consumption and designed specifically for use in NDIR CO_2_ sensors that require signal demodulation. This study used the PULPino SoC platform to implement the five processors and simulate their energy consumption. The results showed that RisCO2 is the most energy-efficient processor, consuming only 0.29 mJ of energy, which is 53.5% and 63.2% less energy than CV32E40P and Ri5cy, respectively. RisCO2 also has the lowest resource utilization compared to the best-performing processors of the study, using only 4889 LUTs, 2354 FFs, and 2 DSPs, which is 46.1%, 7.8%, and 71.4% less than CV32E40P, respectively.

Our experiments show that RisCO2 is a promising candidate for low-power embedded systems that require efficient processing in complex applications with limited hardware resources. The energy-efficient design of RisCO2 also makes it suitable for battery-powered devices, where minimizing energy consumption is critical.

### Future Work

There is potential for improving the performance of RisCO2. The results demonstrate that the instruction throughput (IPC) of RisCO2 is 50% worse than CV32E40P, despite RisCO2 having a deeper pipeline (one more stage) and using the same FPU as CV32E40P. However, increasing the number of pipeline stages can also lead to pipeline hazards and pipeline stalls, decreasing the processor’s overall performance. This issue should be further studied and improved.

Another potential area for improvement is the addition of custom instructions to support hardware loops, a feature that is already present in CV32E40P and Ri5cy. Hardware loops have zero stall cycles for jumping to the first instruction of a loop, which could reduce the runtime of the demodulation algorithm since it involves an iterative process with a large number of iterations equal to the number of samples of the modulated signal. However, the addition of these instructions could increase the hardware complexity of the processor and potentially hinder energy consumption savings. Additionally, modifying the compiler is necessary to generate code that uses the new opcodes.

## 6. Conclusions

In conclusion, the experimental results presented in this study demonstrate the efficiency of the RISC-V architecture for low-power applications, particularly in the context of signal demodulation for NDIR CO_2_ sensors. Our RisCO2 processor design, optimized for energy consumption, showed a significant reduction in energy consumption compared to Ri5cy and CV32E40P while still maintaining competitive performance levels. RisCO2 is a promising candidate for low-power embedded systems that require efficient processing in complex applications with limited hardware resources.

Our results also highlight the importance of considering FPGA resource utilization in designing low-power processors, as it can significantly impact the feasibility of the implementations on resource-limited hardware platforms. Our findings have important implications for the development of energy-efficient processors for low-power applications, especially those requiring real-time signal processing. The results of this study provide a strong foundation for future research in this area, with potential applications in the development of low-power processors for a variety of fields, such as IoT, wearables, and mobile devices. Furthermore, the experimental methodology presented in this study, including the use of switching activity files for power simulation and FPGA implementation, can serve as a valuable reference for other researchers in this area.

Overall, this study contributes to the ongoing efforts to improve the energy efficiency of processors and promote sustainable computing. With the increasing demand for low-power devices in various fields, developing energy-efficient processors is becoming more critical than ever. Our study shows that the RISC-V architecture can offer promising solutions for these challenges, and we hope that our findings will inspire further research and development in this direction.

## Figures and Tables

**Table 1:** Summary of improvements in RisCO2 in terms of FPGA resource utilization, demodulation algorithm runtime, and total energy consumption.

Iteration ^1^	ISA	LUT (% ^2^)	FF (% ^2^)	DSP (% ^2^)	Clock [MHz]	Time (% ^2^) [ms]	Energy (% ^2^) [mJ]
Initial [14]	RV32IMF	7085	4188	12	50	12.2	0.45
1, 2	RV32EM_Zfinx	6518 (0.92)	2693 (0.64)	12 (1.0)	50	10.3 (0.84)	0.42 (0.93)
3	RV32E_Zfinx	5307 (0.75)	2545 (0.61)	2 (0.17)	50	10.3 (0.84)	0.32 (0.71)
5	RV32E_Zfinx	5126 (0.72)	2478 (0.59)	2 (0.17)	50	10.3 (0.84)	0.31 (0.69)
4 (RisCO2)	RV32E_Zfinx	4692 (0.66)	2293 (0.55)	2 (0.17)	50	10.3 (0.84)	0.28 (0.62)

^1^ 1–5 are the items listed in Section 3. ^2^ % is calculated as the factor between the current result and the initial one.

**Table 2:** Comparison of RisCO2 with other RISC-V reference processors in terms of FPGA resource utilization and total energy consumed by the processor for a specific application.

Core	ISA	PipelineStages	LUT	FF	DSP	Var.Type	#instr × 10^6^	IPC	Exec. [ms]	Power [mW]	Energy [mJ]
Micro-riscy	RV32E	2	2225	1276	0	Fixed-point	7.24	0.78	373.13	15	5.60
Zero-riscy	RV32IM	2	3171	1928	1	Fixed-point	1.38	0.82	67.71	20	1.35
Ri5cy	RV32IMF	4	11,912	4249	8	SP Floating	0.28	0.74	15.15	52	0.79
CV32E40P	RV32IMF_Zfinx	4	9072	2553	7	SP Floating	0.25	0.79	12.73	49	0.62
RisCO2	RV32E_Zfinx	5	4889	2354	2	SP Floating	0.25	0.49	20.71	14	0.29

## Data Availability

Data are available on request from the authors.

## Abbreviations

The following abbreviations are used in this manuscript:

ALU	Arithmetic-logic unit
BRAM	Block RAM
CMOS	Complementary metal–oxide–semiconductor
CO_2_	Carbon dioxide
DSP	Digital signal processor
FF	Flip-flop
FPGA	Field-programmable gate array
FPU	Floating-point unit
IDE	Integrated development environment
IoT	Internet of Things
IP	Intellectual property
ISA	Instruction set architecture
IPC	Instruction per cycle
LUT	Lookup table
NDIR	Nondispersive infrared
ppm	Parts per million
ROM	Read-only memory
SAIF	Switching Activity Interchange Format
SoC	System on chip
